# Capsaicin from chili ( *Capsicum* spp.) inhibits vascular smooth muscle cell proliferation

**DOI:** 10.12688/f1000research.6007.1

**Published:** 2015-01-27

**Authors:** Rongxia Liu, Elke H. Heiss, Dean Guo, Verena M. Dirsch, Atanas G. Atanasov

**Affiliations:** 1 Department of Pharmacognosy, University of Vienna, Vienna, Austria; 2 Shanghai Research Center for Modernization of Traditional Chinese Medicine, National Engineering Laboratory for TCM Standardization Technology, Shanghai Institute of Materia Medica, Shanghai Institutes for Biological Sciences, Chinese Academy of Sciences, Shanghai, China

**Keywords:** Capsaicin, vascular smooth muscle cells, restenosis, proliferation

## Abstract

Accelerated vascular smooth muscle cell (VSMC) proliferation is implied in cardiovascular disease and significantly contributes to vessel lumen reduction following surgical interventions such as percutaneous transluminal coronary angioplasty or bypass surgery. Therefore, identification and characterization of compounds and mechanisms able to counteract VSMC proliferation is of potential therapeutic relevance. This work reveals the anti-proliferative effect of the natural product capsaicin from *Capsicum* spp. by quantification of metabolic activity and DNA synthesis in activated VSMC. The observed *in vitro* activity profile of capsaicin warrants further research on its mechanism of action and potential for therapeutic application.

## Main text

Aberrant and accelerated VSMC proliferation is a main contributor to restenosis, the pathological re-narrowing of the vessel lumen after surgical interventions combating vascular stenosis. To overcome restenosis, drug-eluting stents have been developed, aiming at inhibiting VSMC growth by the release of anti-proliferative substances such as paclitaxel and rapamycin. However, these compounds display unresolved issues such as impaired re-endothelialization and subsequent thrombosis induction ^1^, which makes the characterization of other compounds able to suppress VSMC proliferation highly relevant. Plant-derived natural products are an excellent resource for identifying lead compounds ^2^. Here we examine the anti-proliferative potential of capsaicin, a bioactive component of chili peppers [ *Capsicum* spp. (Solanaceae)], in VSMC.

**Figure 1.**
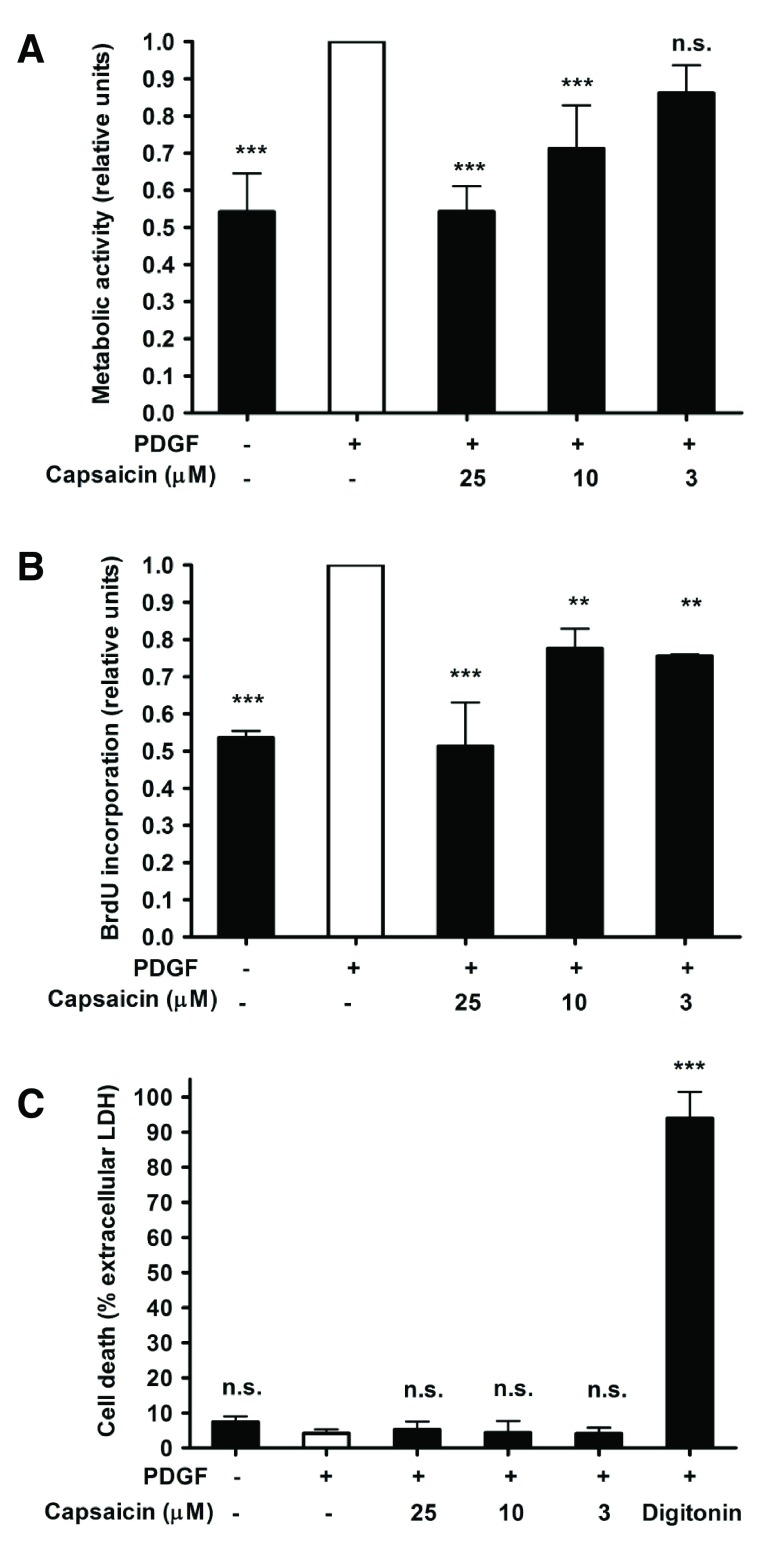
Effect of capsaicin on VSMC proliferation. Cell proliferation was estimated by quantification of metabolic activity ( **A**) and DNA synthesis ( **B**). Cell death was estimated by quantification of the percentage of extracellular LDH ( **C**). Data represent mean ± SD from at least three independent experiments (n.s., not significant; ***p < 0.001; **p < 0.01; ANOVA/Bonferroni).

To test whether capsaicin is able to inhibit proliferation of VSMC induced by PDGF, a major growth factor implied in the aberrant proliferative responses in restenosis ^3^, the total amount of metabolically active cells was measured after 48 h of incubation by the resazurin conversion method ^4^. Capsaicin indeed suppressed VSMC proliferation concentration-dependently with an IC _50_ of 5.36 μM ( Figure 1A). To confirm the anti-proliferative effect of capsaicin with a second experimental method, we measured DNA synthesis in VSMC by quantification of 5-bromo-2′-deoxyuridine (BrdU) incorporation into DNA. Capsaicin also inhibited PDGF-stimulated DNA synthesis in a concentration-dependent manner with an IC _50_ of 3.81 μM ( Figure 1B). To assure that the decreased number of VSMC upon treatment with capsaicin is not due to cytotoxicity, we quantified cell death by measuring cell membrane integrity estimated by lactate dehydrogenase (LDH) activity inside cells and in cell supernatants. No significant cytotoxicity was detected in the investigated concentration range ( Figure 1C). In summary, capsaicin is identified as an inhibitor of VSMC proliferation. Further studies are prompted to elaborate the underlying mode of action of this natural product and to investigate its effect in advanced *in vivo* anti-restenotic models.

Rat aortic VSMC used in this study were purchased from Lonza (Braine-L’Alleud, Belgium) and cultivated in DMEM–F12 (1:1) medium supplemented with 20% fetal calf serum and gentamycin. Capsaicin and other chemicals were obtained from Sigma-Aldrich (Vienna, Austria).

For the resazurin conversion assay, VSMC were seeded in 96-well plates at 5 × 10 ^3^ cells/well. 24 h later, cells were serum-starved for 24 h to render them quiescent. Quiescent cells were pretreated for 30 min with capsaicin or vehicle (0.1% DMSO) as indicated, and subsequently stimulated for 48 h with PDGF-BB (20 ng/mL). To measure the number of metabolically active VSMC by resazurin conversion ^4^, cells were washed with PBS and incubated in serum-free medium containing 10 μg/mL resazurin for 2 h. Total metabolic activity was measured by monitoring the increase in fluorescence at a wavelength of 590 nm using an excitation wavelength of 535 nm in a 96-well plate reader (Tecan GENios Pro).

For the BrdU incorporation assay, VSMC were seeded and starved as for the resazurin conversion assay. Quiescent cells were pretreated for 30 min with capsaicin, or vehicle as indicated and subsequently stimulated with PDGF-BB (20 ng/mL). To estimate *de novo* DNA synthesis in VSMC ^5^, BrdU was added 2 h after PDGF stimulation, and the incorporated amount was determined 22 h afterwards with a BrdU ELISA kit according to the manufacturer’s instructions (Roche Diagnostics).

For assessing cytotoxicity, VSMC were seeded and serum-starved as indicated above. The quiescent cells were pretreated for 30 min with capsaicin, or vehicle as indicated, and subsequently stimulated for 24 h with PDGF-BB (20 ng/mL). To quantify the loss of cell membrane integrity as a sign for cell death ^6^, the supernatants of the treated cells were assessed for LDH activity. For estimation of the total LDH, identically treated samples were incubated for 45 min in the presence of 1% Triton X-100. The released and total LDH enzyme activity was quantified for 30 min in the dark in the presence of 4.5 mg/mL lactate, 0.56 mg/mL NAD+, 1.69 U/mL diaphorase, 0.004% (w/v) BSA, 0.15% (w/v) sucrose, and 0.5 mM 2-p-iodophenyl-3-nitrophenyl tetrazolium chloride (INT). The enzyme reaction was stopped with 1.78 mg/mL oxymate and the absorbance was measured at 490 nm in a 96-well plate reader (Tecan GENios Pro). Potential effects on cell viability were estimated as percentage of extracellular LDH activity. The cytotoxic natural product digitonin (100 μg/mL) was used as a positive control.

Statistical analysis was performed by ANOVA/Bonferroni test (GraphPad PRISM software, version 4).

Effect of capsaicin on vascular smooth muscle cell proliferation: raw data Cell proliferation was estimated by quantification of metabolic activity (Data_1A) and DNA synthesis (Data_1B) (both relative units). Cell death was estimated by quantification of the percentage of extracellular lactate dehydrogenase activity (Data_1C). Numbers in column headers denote capsaicin concentration (uM); Digitonin concentration was 100 ug/mL). Data correspond to Figure 1 in the associated article.

## Data availability

*Figshare:* Effect of capsaicin on vascular smooth muscle cell proliferation: raw data. doi: 10.6084/m9.figshare.1289721 ^7^
