# Supplementary material for: Capsaicin from chili ( Capsicum spp.) inhibits vascular smooth muscle cell proliferation
Source: F1000Res. 2015 Jan 27;4:26. [Version 1] doi: 10.12688/f1000research.6007.1 (PMC4314658; doi:10.12688/f1000research.6007.1)
Supplement: Peer review history

# Peer review: Referee response for version 1

**DOI:** 10.5256/f1000research.6428.r7485

**Published:** 2015-02-02

**Authors:** Karel Šmejkal

**Affiliations:** 1 Department of Natural Drugs, University of Veterinary and Pharmaceutical Sciences, Brno, Czech Republic

The authors are describing the inhibitory activity of capsaicin on vascular smooth muscle cell proliferation. The article looks to be well written, with adequate data. I miss only a bit deeper discussion of reasons for capsaicin selection for testing.

I have read this submission. I believe that I have an appropriate level of expertise to confirm that it is of an acceptable scientific standard.

# Peer review: Referee response for version 1

**DOI:** 10.5256/f1000research.6428.r7484

**Published:** 2015-02-02

**Authors:** Goutam Brahmachari

**Affiliations:** 1 Laboratory of Natural Products & Organic Synthesis, Department of Chemistry, Visva-Bharati University, Santiniketan, West Bengal, India

I have gone through the present manuscript entitled “Capsaicin from chili ( *Capsicum *spp.) inhibits vascular smooth muscle cell proliferation” by Liu *et al*. with interest where the investigators demonstrated the efficacy of natural capsaicin in overcoming restenosis by using a vascular muscle cell model (VSMC) *in vitro*.

Besides macrophages, vascular smooth muscle cells (VSMCs) are now also believed to play a significant role in generating foam cells that accumulate cytoplasmic droplets of cholesterol esters and triglycerides leading to atherosclerosis regarded as a prime cause of cardio- and cerebrovascular events. Proliferation of VSMCs thus is a common cause of restenosis among the patients who were previously undergone percutaneous transluminal coronary angioplasty or bypass surgery. Under this purview, the present work demonstrating promising *in vitro* anti-proliferative activity of natural capsaicin seems to be much interesting.

The experiments are straightforward and sufficient in arriving at the conclusions. Representation of facts and organization of the manuscript are praise-worthy. The manuscript may be accepted and indexed in its present form. At the same time, I have a suggestion for the investigators as mentioned below, which may be addressed to make the revised manuscript more demanding.

**Suggestion**

Rapamycin and paclitaxel are commonly used standard drugs to resist restenosis in patients although they suffer from certain serious health issues as mentioned by the authors in their present manuscript. Hence, search for better alternative(s) is warranted as a part of which the present work has been developed. Hence, I do suggest comparing the present results with those of at least any one of these two drugs (in addition to or in place of digitonin) to validate superiority of capsaicin.

I have read this submission. I believe that I have an appropriate level of expertise to confirm that it is of an acceptable scientific standard.

# Peer review: Referee response for version 1

**DOI:** 10.5256/f1000research.6428.r7483

**Published:** 2015-01-30

**Authors:** Carsten Gründemann

**Affiliations:** 1 Center for Complementary Medicine, Universitätsklinikum Freiburg, Freiburg, Germany

In the current manuscript, Rongxia Liu *et al *investigated the influence of the plant-derived natural product capsaicin to overcome restenosis by using a vascular muscle cell model

(VSCM) *in vitro*.

The manuscript is well written and conclusion is precise. The experiments are straightforward and the presented cell-based model is adequate to investigate such question. Nevertheless, before indexation the comments below have to be improved in a revised version of the manuscript.

**Major Comment:**

**Main text:** The authors mentioned that paclitaxel and rapamycin are used as standard drugs to inhibit VSCM proliferation. For pharmacological testing, it is essential that the authors should compare the investigated capsaicin to a standard therapy (positive control) to get an idea about capsaicin’s bio-activity.

**Minor Comment:**

**Abstract: **The authors have to depict more clearly why it is important to investigate new natural-derived compounds to overcome stenosis.

I have read this submission. I believe that I have an appropriate level of expertise to confirm that it is of an acceptable scientific standard, however I have significant reservations, as outlined above.
